# Vagus Nerve Stimulation Regulates the Th17/Treg Balance and Alleviates Lung Injury in Acute Respiratory Distress Syndrome by Upregulating α7nAChR

**DOI:** 10.3390/biomedicines13061294

**Published:** 2025-05-24

**Authors:** Furong Zheng, Xin Zhang, Sisi Wang, Gongwei Jia, Li Cheng

**Affiliations:** 1 Department of Rehabilitation, The Second Affiliated Hospital of Chongqing Medical University, Chongqing 400010, China; 2022110370@stu.cqmu.edu.cn (F.Z.); 2021110393@stu.cqmu.edu.cn (X.Z.); 2022110367@stu.cqmu.edu.cn (S.W.); 2 Department of Health Management Center, The Second Affiliated Hospital of Chongqing Medical University, Chongqing 400010, China

**Keywords:** ARDS, VNS, Th17, Treg, α7nAChR

## Abstract

**Background:** Acute respiratory distress syndrome (ARDS) is a high-mortality disease strongly associated with an imbalance in the inflammatory response. The ratio of helper T 17 (Th17) cells to regulatory T (Treg) cells is significantly correlated with prognosis and outcomes in ARDS. Vagus nerve stimulation (VNS) alleviates lung injury in ARDS model rats. The objective of this study was to further investigate whether VNS attenuates lipopolysaccharide-induced ARDS by regulating Th17/Treg homeostasis and to explore the underlying mechanisms. **Methods**: We assessed the degree of lung injury using hematoxylin and eosin staining, the lung wet-to-dry ratio, and total protein and pro-inflammatory cytokine levels in bronchoalveolar lavage fluid. The expression levels of Th17 and Treg cells were determined using flow cytometry, Western blotting, quantitative real-time PCR, and enzyme-linked immunosorbent assays. **Results**: We found that VNS reduced lung injury in ARDS model rats. Additionally, VNS regulated Th17/Treg homeostasis and reduced the levels of inflammatory factors in both the lungs and spleens. Notably, the effects of VNS were consistent when the afferent or efferent vagus nerve, or both, were stimulated. Further investigation revealed that VNS upregulated splenic α7 nicotinic acetylcholine receptors (α7nAChRs). The administration of an α7nAChR agonist enhanced VNS-mediated regulation of Th17/Treg homeostasis and attenuated lung injury, while these effects were blocked by α7nAChR antagonists. **Conclusions**: Our study demonstrated that VNS regulates the Th17/Treg balance through α7nAChR activation in the spleen, thereby mitigating lung injury in ARDS. These findings provide new theoretical support for the use of VNS in attenuating ARDS.

## 1. Introduction

Acute respiratory distress syndrome (ARDS) is a fulminant disease with a mortality rate exceeding 40% that is characterized by acute, diffuse, and severe inflammatory injury to the lungs [1,2]. The pathogenesis of ARDS remains incompletely understood, but studies have demonstrated that the activation of multiple inflammatory cells and the release of inflammatory cytokines serve as key factors in its development and outcome [3]. The excessive recruitment and activation of inflammatory cells leads to an imbalance in the inflammatory response within the lungs and even throughout the body. Therefore, regulating the balance of the inflammatory response is a central focus in ARDS treatment.

The vagus nerve can sense inflammation and modulate the inflammatory response by regulating key immune organs [4]. The cholinergic anti-inflammatory pathway (CAIP) and the hypothalamic–pituitary–adrenal (HPA) axis are the two primary anti-inflammatory pathways of action of vagus nerve stimulation (VNS). CAIP primarily involves the activation of α7 nicotinic acetylcholine receptors (α7nAChRs) on macrophages by vagal efferent fibers, leading to the inhibition of pro-inflammatory cytokine release and subsequent attenuation of inflammation [5,6]. In rat models of ARDS, VNS modulates the inflammatory response by decreasing pro-inflammatory cytokines (e.g., TNF-α and IL-1β) and enhancing anti-inflammatory cytokines (e.g., IL-10), thereby mitigating lung injury [7]. However, the precise mechanisms underlying these effects have not yet been fully elucidated. The HPA axis is primarily activated when vagal afferent fibers detect IL-1β, transmitting the signal via the nucleus tractus solitarius to the paraventricular nucleus of the hypothalamus, which then stimulates the pituitary–adrenal axis to promote the synthesis and release of glucocorticoids, thereby suppressing pro-inflammatory cytokines and resolving inflammation [8]. Clinical studies suggest that glucocorticoids may ameliorate the severity of ARDS [9]. However, whether VNS modulates the pulmonary inflammatory response in ARDS through HPA axis-mediated anti-inflammatory pathways remains to be clarified.

In recent years, studies have shown that the immune response mediated by CD4-positive T lymphocyte subsets, including T helper 17 (Th17) cells and regulatory T (Treg) cells, significantly influences the severity of inflammation in ARDS, with Th17 and Treg cells exerting opposing effects [10,11,12]. The differentiation of CD4-positive T cells into Th17 cells is driven by the synergistic action of cytokines such as TGF-β, IL-6, and IL-23, along with the transcription factor RORγt [13,14,15]. Activated Th17 cells exacerbate inflammation and lung injury by secreting IL-17 and various chemokines, which recruit neutrophils and amplify the production of pro-inflammation cytokines [11,16]. Conversely, during ARDS progression, CD4-positive T cells differentiate into Treg cells in response to TGF-β, IL-2, and the transcription factor FOXP3 [15,17,18]. Treg cells secrete the anti-inflammatory cytokine IL-10, which enhances alveolar neutrophil clearance and regulates macrophage activity, thereby modulating the innate immune response, reducing lung injury, and improving the survival rate [12,19]. Clinical studies have reported that the Th17/Treg ratio in peripheral blood increases 24 h after the onset of ARDS and is positively correlated with the 28-day mortality rate [20]. Therefore, we believe that maintaining the balance between Th17 and Treg cells may be critical for resolving inflammation and attenuating the immune cascade response in ARDS. In the hemorrhagic shock model, VNS inhibits Th17 cell differentiation and promotes Treg cell differentiation, thereby affecting Th17/Treg homeostasis [21]. Therefore, we aim to investigate whether the mechanism by which VNS alleviates the inflammatory response in ARDS is related to Th17/Treg homeostasis.

In this study, an LPS-induced ARDS rat model was established to systematically investigate the regulatory effects of intact, afferent, and efferent VNS on Th17/Treg homeostasis and inflammatory cytokine dynamics. Mechanistic studies were subsequently conducted, using adrenalectomy and splenectomy to delineate the neural pathways underlying VNS-mediated effects. Pharmacological interventions using α7nAChR agonists and antagonists were performed to elucidate the molecular mechanisms underlying VNS-mediated immunomodulation. Collectively, these findings establish a mechanistic framework demonstrating that VNS ameliorates LPS-induced lung injury via α7nAChR-dependent regulation of Th17/Treg homeostasis, thereby providing a theoretical basis for the development of neuromodulation-based therapeutic strategies for ARDS.

## 2. Materials and Methods

### 2.1. Animals

Healthy adult male Sprague-Dawley rats (200–250 g) were purchased from the Laboratory Animal Center of Chongqing Medical University (Chongqing, China). The rats were housed under a 12-h light/dark cycle at a temperature of 21–22 °C and relative humidity of 60%, with unrestricted access to food and water. The experimental protocols were approved by the Ethics Committee for Animal Experiments and the Institutional Animal Care and Use Committee of the Second Affiliated Hospital of Chongqing Medical University (Approval No. IACUC-SAHCQMU-2023-0034).

### 2.2. ARDS Model

The ARDS model was established according to the previously reported methodology [22]. Rats were anaesthetized with 3% sodium pentobarbital and administered a dose of 2 mg/kg of LPS (*Escherichia coli* LPS serotype 0111:B4 dissolved in phosphate-buffered saline (PBS) using a tracheal drip). The specific experimental groups were as follows: control group (rats received intratracheal instillation of saline; *n* = 6); LPS group (rats received intratracheal instillation of LPS; *n* = 9); LPS + VNS group (rats underwent VNS 6 h after intratracheal LPS instillation; *n* = 12); ADX + LPS + VNS group (rats underwent adrenalectomy 7 days before intratracheal instillation of LPS, followed by VNS 6 h after intratracheal instillation of LPS; *n* = 6); SPX + LPS + VNS group (rats underwent splenectomy 7 days before intratracheal instillation of LPS, followed by VNS 6 h after intratracheal instillation of LPS; *n* = 6); GTS-21 + LPS + VNS group (rats received an intraperitoneal injection of the α7nAChR agonist GTS-21 30 min before intratracheal instillation of LPS, followed by VNS 6 h after intratracheal instillation of LPS; *n* = 6); and MLA + LPS + VNS group (rats received intraperitoneal injection of the α7nAChR antagonist MLA 30 min before intratracheal instillation of LPS, followed by VNS 6 h after intratracheal instillation of LPS; *n* = 6). Rats were euthanized 8 h after LPS administration, and samples were collected for experimental analysis.

### 2.3. Electrical Stimulation of the Vagus Nerve

Rats were anaesthetized, and a median cervico-ventral incision was made to expose the salivary glands. The glands were retracted laterally to visualize the left carotid artery and isolate the vagus nerve. The nerve was then positioned on a bipolar hooked electrode connected to an electrical stimulation device. In the vagus nerve stimulation (VNS) group, rats received stimulation at 5 volts, 5 hertz, and 2 milliseconds for a period of 10 min, administered 6 h after ARDS model induction [23]. Following the surgical procedure, the cervical incision was sutured, and the rats were maintained at a constant temperature using an insulating pad and closely monitored until full recovery from anesthesia. In the control group, the vagus nerve was exposed but not stimulated.

### 2.4. Splenectomy

Rats were anaesthetized, and a left abdominal incision was made. The splenic vessels were ligated, and the spleen was removed. Then, the wound was sutured. Rats were allowed to recover for 7 days.

### 2.5. Adrenalectomy

Rats were anaesthetized as previously described, and a dorsal incision was made to remove both adrenal glands, followed by suturing of the incision. Adrenalectomized rats were provided free access to 0.9% NaCl solution for drinking.

### 2.6. Administration of α7nAChR Agonist and Antagonist

The α7nAChR agonist GTS-21 (4 mg/kg, MCE, Monmouth, NJ, USA) and antagonist MLA (6 mg/kg, MCE, Monmouth, NJ, USA) were used to modulate α7nAChR activity [24,25]. According to previous research methods, GTS-21 and MLA were diluted in PBS and administered intraperitoneally 30 min prior to LPS infusion.

### 2.7. H&E Staining

The upper lobe of the right lung was harvested, fixed in 4% paraformaldehyde, embedded in paraffin, sectioned at 5 µm thickness, and stained with hematoxylin and eosin (H&E). Histological evaluation was performed under a light microscope by analyzing 10 randomly selected high-power fields (HPFs; ×100) per section. Lung injury was scored according to Szapiel’s criteria, which included four categories: 0, normal lung morphology; 1, inflammatory damage < 20%; 2, inflammatory damage 20–50%; and 3, inflammatory damage > 50% [26]. Lung injury scores were assessed by pathologists blinded to the experimental groups.

### 2.8. Lung Wet-to-Dry Ratio (W/D)

The middle lobe of the right lung was excised, weighed, and dried in an oven at 55 °C for 72 h. The dry weight was then measured, and the wet-to-dry (W/D) ratio was calculated.

### 2.9. Bronchoalveolar Lavage Fluid (BALF)

The alveolar spaces were lavaged three times with 2 mL of PBS each time via tracheal intubation, and over 80% of the instilled volume was recovered as bronchoalveolar lavage fluid (BALF). The supernatant was obtained by centrifugation at 500× *g* for 20 min at 4 °C and stored at −80 °C for further analysis.

### 2.10. Enzyme-Linked Immunosorbent Assay (ELISA)

The levels of cytokines, including tumor necrosis factor-α (TNF-α), interleukin-1β (IL-1β), interleukin-2 (IL-2), interleukin-6 (IL-6), interleukin-10 (IL-10), interleukin-17 (IL-17), and interleukin-23 (IL-23), were measured in the collected BALF using ELISA kits (Boster and Fine Test, Wuhan, China), according to the manufacturer’s instructions.

### 2.11. Western Blotting (WB)

Proteins from spleen and lung tissues were extracted using a whole protein extraction kit (Beyotime, Shanghai, China), followed by protein concentration determination using BCA protein assay reagent (Beyotime, China). After boiling and denaturation, the proteins were stored at −80 °C until further analysis. Equal amounts of protein were loaded into each well, separated using SDS-PAGE on a 7.5% separating gel, and transferred to a PVDF membrane. The membrane was then blocked with 5% skimmed milk for one hour at room temperature. Subsequently, the membrane was incubated overnight at 4 °C with the following primary antibodies: anti-RORγt (1:1000, WL05118, Wanleibio, Shenyang, China), anti-FOXP3 (1:1000, WL00721, Wanleibio, China), anti-CHRNA7 (1:1000, bs-1049R, Bioss, Beijing, China), and anti-GAPDH (1:5000, 60004-1-Ig, Proteintech, Wuhan, China). GAPDH was used as a loading control.

### 2.12. Flow Cytometry

To prepare a single-cell suspension, spleen and lung tissues were minced, homogenized, and passed through a 70 μm nylon cell strainer to remove tissue debris. The resulting cell suspension was treated with erythrocyte lysis buffer and resuspended in PBS. Surface proteins were first immunolabelled with FITC-labelled anti-CD4 (11-0040-81, Invitrogen, Carlsbad, CA, USA) and PE-labelled anti-CD25 (12-0390-82, Invitrogen, USA). Cells were then fixed and permeabilized using the FOXP3/Transcription Factor Staining Buffer Set (00-5523-00, Invitrogen, USA). Finally, the intracellular proteins were stained with PE-labelled anti-IL-17A (12-7177-81, Invitrogen, USA) and APC-labelled anti-FOXP3 (17-5773-82, Invitrogen, USA). The stained cells were collected and analyzed on a flow cytometer (Beckman Coulter, Pasadena, CA, USA), and data were analyzed using the FlowJo 10.8.1 software (BD Biosciences, Franklin Lakes, NJ, USA).

### 2.13. RNA Isolation and Quantitative Real-Time PCR (qRT-PCR)

Total RNA was extracted from homogenized lung and spleen tissues using the SteadyPure Rapid RNA Extraction Kit (Accurate Bio, Changsha, China). Complementary DNA (cDNA) was subsequently synthesized by reverse transcription using the PrimeScript RT Kit (Takara Bio Inc., Kyoto, Japan), followed by quantitative real-time PCR (qRT-PCR) using the TB Green Premix Ex Taq II (Takara Bio, Inc.). Relative gene expression levels were normalized to that of the endogenous control GAPDH using the comparative Ct (ΔΔCt) method. The Gene IDs were as follows: RORγt (368158), FOXP3 (317382), and GAPDH (24383). Primers were designed based on the following criteria: an amplification product size of 80–150 base pairs, primer length of 18–23 base, base G and C content between 40% and 60%, and temperature (Tm) ranging from 55 °C to 60 °C. The base sequences were randomly distributed, and all primers were verified for specificity and designed to minimize self-complementarity and complementarity between primer pairs. The sequences of the primers have been designed as follows: RORγt, 5′-CAGAGATGCTGTCAAGTTCGG-3′ (forward primer) and 5′-GCTACTTGTTCCCGTTGCTG-3′ (reverse primer); FOXP3, 5′-CCTGCCACCTGGGATCAATG-3′ (forward primer) and 5′-CCTGCCACCTGGGATCAAT G-3′ (reverse primer); and GAPDH, 5′-GGCACAGTCAAGGCTGAGAAT-3′ (forward primer) and 5′-ATGGTGGTGAAGACGCCAGTA-3′ (reverse primer).

### 2.14. Statistical Analysis

All data are presented as mean ± standard deviation (SD) and were analyzed using GraphPad Prism 8.0.2 (San Diego, CA, USA). One-way analysis of variance (ANOVA) followed by Bonferroni’s post-hoc test was used for multiple group comparisons. Numerical differences were considered significant when *p* < 0.05.

## 3. Results

### 3.1. VNS Regulates Th17/Treg Homeostasis and Reduces Lung Inflammation in ARDS Model Rats

**Figure 1:**
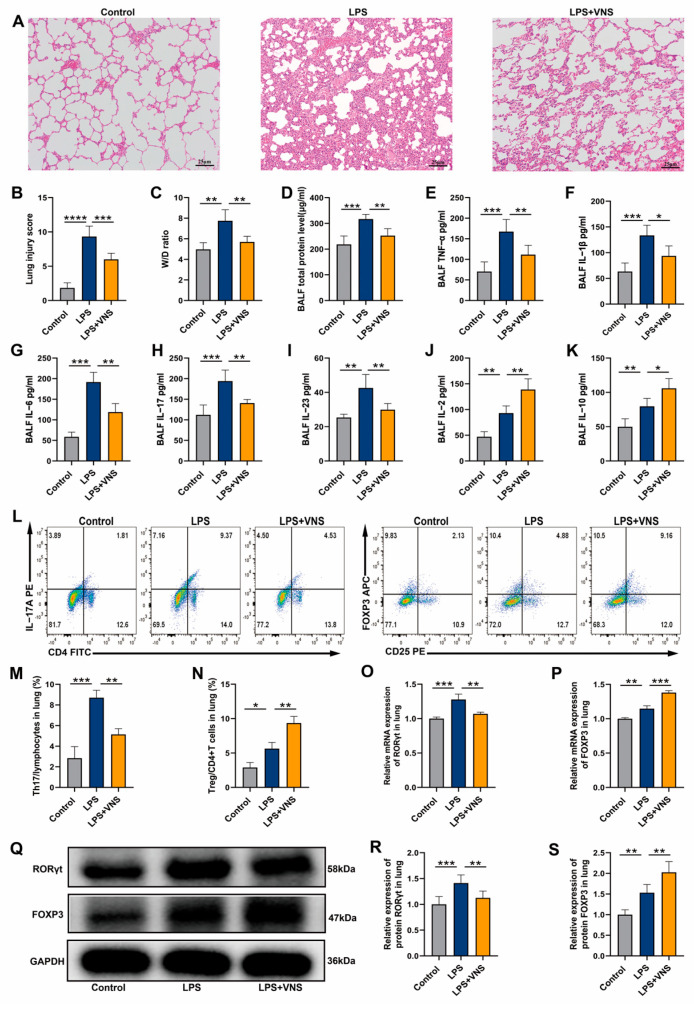
(**A**) Representative images of H&E staining (100× magnification) of lung tissue sections from each group. (**B**) Lung injury score analysis. (**C**) The wet-to-dry ratio of lung tissues. (**D**) Protein concentrations in BALF. (**E**–**K**) The levels of inflammatory cytokines TNF-α, IL-1β, IL-6, IL-17, IL-23, IL-2, and IL-10 in BALF as assessed with ELISA. (**L**–**N**) The percentages of CD4+ IL-17A+ T cells and CD4+ CD25+ FOXP3+ T cells in lung tissues from each group were determined using flow cytometry. (**O**,**P**) RORγt and FOXP3 mRNA levels in lung tissues were measured using qRT-PCR. (**Q**–**S**) RORγt and FOXP3 expression levels in lung tissue were analyzed using Western blots. Data are presented as means ± SDs and analyzed using one-way ANOVA followed by the Bonferroni’s post-hoc test. *n* = 3–6 for each group. * *p* < 0.05, ** *p* < 0.01, and *** *p* < 0.001, **** *p* < 0.0001 indicate significant differences from each group.

We initially investigated the effects of VNS on lung injury in ARDS. H&E staining was used to assess the structural changes in lung tissue. After the LPS tracheal drip, the lungs displayed marked thickening of the alveolar walls, narrowing of the alveolar spaces, increased inflammatory cell infiltration, and elevated lung injury scores (Figure 1A,B). However, VNS treatment significantly ameliorated these pathological alterations and reduced the lung injury scores (Figure 1A,B). Additionally, VNS treatment significantly improved LPS-induced pulmonary edema, as evidenced by a reduction in the wet-to-dry ratio (W/D) (Figure 1C). We further assessed the regulatory effects of VNS on inflammatory cytokines and Th17/Treg homeostasis in the BALF and lung tissues of ARDS model rats. The results showed that VNS significantly reduced total protein levels, as well as the levels of pro-inflammatory cytokines TNF-α and IL-1β in the BALF of LPS-induced ARDS rats (Figure 1D–F). Further analysis revealed that VNS reduced the levels of Th17-related cytokines IL-6, IL-17, and IL-23 in BALF (Figure 1G–I) but increased the levels of Treg-related cytokines IL-2 and IL-10 (Figure 1J,K). Additionally, VNS treatment led to a reduction in the percentage of Th17 cells, an increase in the percentage of Treg cells, and a decrease in the Th17/Treg ratio in lung tissues compared to the LPS group (Figure 1L–N). Gene and protein expression analysis of Th17 and Treg transcription factors in lung tissues using qRT-PCR and Western blot showed that VNS significantly decreased RORγt expression and increased FOXP3 expression compared to the LPS group (Figure 1O–S). These findings suggest that VNS effectively attenuates lung injury, reduces inflammation, and restores Th17/Treg homeostasis in ARDS model rats.

### 3.2. Afferent and Efferent VNS Regulates Th17/Treg Homeostasis and Reduces Lung Inflammation in ARDS Model Rats

**Figure 2:**
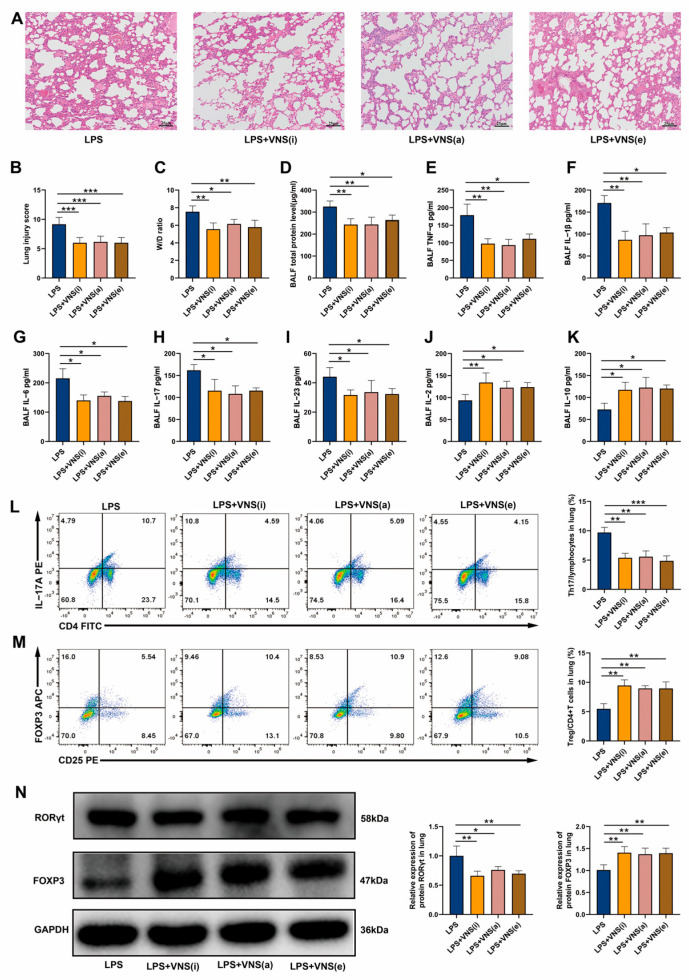
(**A**) Representative images of H&E staining (100× magnification) of lung tissue sections from each group. (**B**) Lung injury score analysis. (**C**) The wet-to-dry ratio of lung tissues. (**D**) Protein concentrations in BALF. (**E**–**K**) The levels of inflammatory cytokines TNF-α, IL-1β, IL-6, IL-17, IL-23, IL-2, and IL-10 in BALF as assessed using ELISA. (**L**,**M**) The percentages of CD4+ IL-17A+ T cells and CD4+ CD25+ FOXP3+ T cells in lung tissues from each group were determined using flow cytometry. (**N**) RORγt and FOXP3 expression in lung tissue was analyzed using Western blots. Data are presented as means ± SDs and analyzed using one-way ANOVA followed by the Bonferroni’s post-hoc test. *n* = 3–6 for each group. * *p* < 0.05, ** *p* < 0.01, and *** *p* < 0.001 indicate significant differences from each group. VNS(i), intact VNS; VNS(a), afferent VNS; VNS(e), efferent VNS.

The vagus nerve is composed of 80% afferent fibers and 20% efferent fibers [27]. The afferent fibers are primarily responsible for transmitting signals to the central nervous system, while the efferent fibers are responsible for regulating peripheral inflammation [27]. To further elucidate the pathway through which VNS modulates Th17/Treg homeostasis and alleviates lung injury, we stimulated the intact left cervical vagus nerve (VNS [i]), the central end of the cut left cervical vagus nerve (VNS [a], afferent), and the peripheral end of the cut left cervical vagus nerve (VNS [e], efferent), separately. We first investigated the effects of afferent and efferent VNS on lung injury. H&E staining results showed that, compared to the LPS group, intact, afferent, and efferent VNS all ameliorated lung injury, reduced lung injury scores, and alleviated pulmonary edema (Figure 2A–C). We further evaluated the effects of afferent and efferent VNS on inflammatory factors and Th17/Treg homeostasis in the BALF and lung tissues of ARDS model rats. The results showed that, consistent with the effects of intact VNS, both afferent and efferent VNS significantly reduced the total protein concentrations and levels of pro-inflammatory cytokines TNF-α and IL-1β in the BALF (Figure 2D–F). Further analysis revealed that, similar to intact VNS, both afferent and efferent VNS decreased the levels of Th17-related cytokines IL-6, IL-17, and IL-23 in the BALF (Figure 2G–I), while increasing the levels of Treg-related cytokines IL-2 and IL-10 (Figure 2J,K). Additionally, intact, afferent, and efferent VNS interventions all reduced the percentage of Th17 cells in lung tissue, increased the percentage of Treg cells, and decreased the Th17/Treg ratio (Figure 2L,M). Western blot analysis revealed that, similar to intact VNS, both afferent and efferent stimulation significantly downregulated RORγt and upregulated FOXP3 expression (Figure 2N). These results suggest that, similar to intact VNS, both afferent and efferent VNS alleviate lung injury, reduce inflammation, and regulate Th17/Treg homeostasis in ARDS model rats.

### 3.3. VNS Modulates Th17/Treg Homeostasis and Attenuates Pulmonary Inflammation Independently of the Adrenal Glands in a Rat Model of ARDS

**Figure 3:**
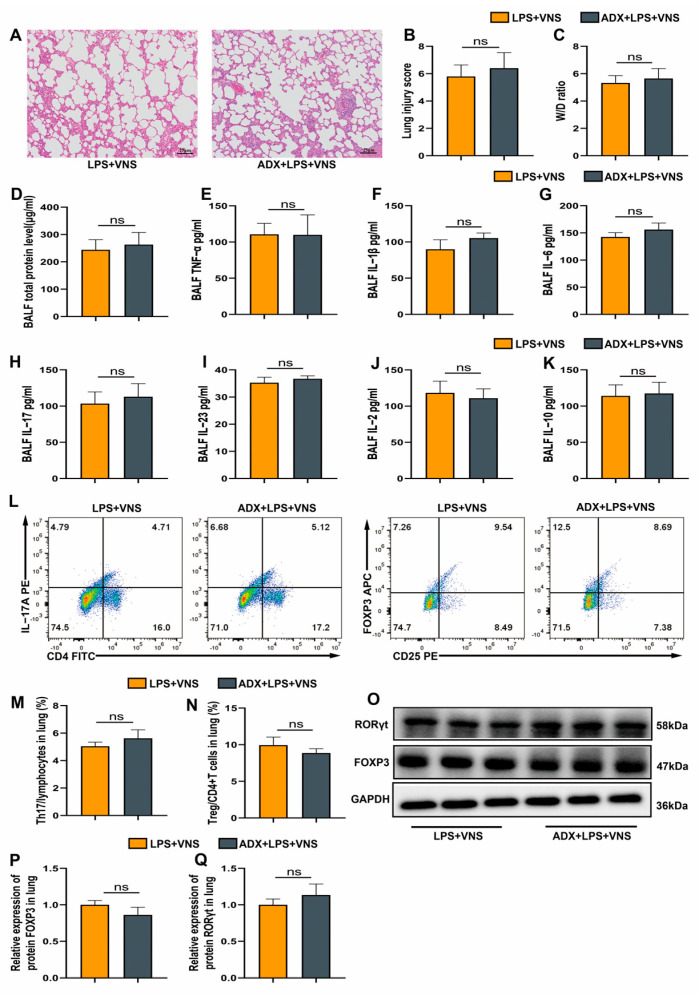
(**A**) Representative images of H&E staining (100× magnification) of lung tissue sections from each group. (**B**) Lung injury score analysis. (**C**) The wet-to-dry ratio of lung tissues. (**D**) Protein concentrations in BALF. (**E**–**K**) The levels of inflammatory cytokines TNF-α, IL-1β, IL-6, IL-17, IL-23, IL-2, an IL-10 in BALF as assessed using ELISA. (**L**–**N**) The percentages of CD4+ IL-17A+ T cells and CD4+ CD25+ FOXP3+ T cells in lung tissues from each group were determined using flow cytometry. (**O**–**Q**) RORγt and FOXP3 expression in lung tissue was analyzed using Western blots. Data are presented as means ± SDs and analyzed using unpaired *T* tests. *n* = 3–6 for each group. ADX, adrenalectomy.

The hypothalamic–pituitary–adrenal (HPA) axis mediates anti-inflammatory effects through the activation of afferent vagal fibers, with the adrenal gland serving as a key node in this pathway [8]. To further investigate the route by which VNS regulates Th17/Treg homeostasis and attenuates the inflammatory response in ARDS, we surgically excised the bilateral adrenal glands of rats 7 days prior to LPS tracheal instillation to assess whether adrenalectomy influences the effects of VNS. Because stimulation of the intact vagus nerve activates both efferent and afferent fibers, we selected this approach for this observation. We first investigated the impact of adrenalectomy on the ability of VNS to alleviate lung injury. Pathological analysis using H&E staining showed that the ADX + LPS + VNS group did not exhibit significantly greater lung histopathological damage compared with the LPS + VNS group, and no significant difference in lung injury scores were noted between the two groups (Figure 3A,B). Similarly, no significant changes were observed in the lung wet-to-dry (W/D) ratio between these groups (Figure 3C). We further investigated whether adrenalectomy influences the anti-inflammatory and immunomodulatory effects of VNS on Th17/Treg homeostasis. Total protein concentrations and pro-inflammatory cytokine levels (TNF-α and IL-1β) in BALF did not significantly differ between the ADX + LPS + VNS and LPS + VNS groups (Figure 3D–F). Moreover, ADX pretreatment did not significantly alter IL-6, IL-17, IL-23, IL-2, or IL-10 levels (Figure 3G–K). Flow cytometric analysis revealed no significant difference in the proportions of Th17 and Treg cells in lung tissues between the two groups (Figure 3L–N). Consistently, the expression of the transcription factors RORγt and FOXP3 were also not altered (Figure 3O–Q). Collectively, these findings suggest that the anti-inflammatory and immunoregulatory effects of VNS in ARDS are independent of the HPA axis. These comprehensive findings indicate that the pathways by which VNS attenuates lung injury, reduces inflammatory responses, and regulates Th17/Treg homeostasis in ARDS model rats occur independently of the HPA axis.

### 3.4. VNS Regulation of Th17/Treg Homeostasis and Attenuation of the Inflammatory Response in ARDS Model Rats Requires Spleen Involvement

**Figure 4:**
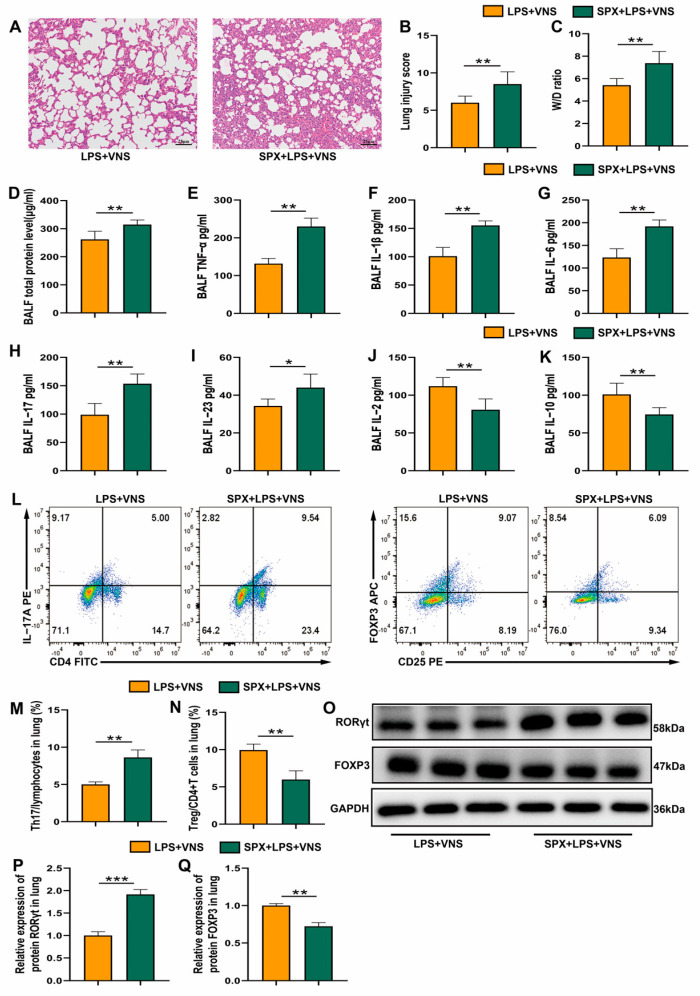
(**A**) Representative images of H&E staining (100× magnification) of lung tissue sections from each group. (**B**) Lung injury score analysis. (**C**) The wet-to-dry ratio of lung tissues. (**D**) Protein concentrations in BALF. (**E**–**K**) The level of inflammatory cytokines TNF-α, IL-1β, IL-6, IL-17, IL-23, IL-2, and IL-10 in BALF as assessed using ELISA. (**L**–**N**) The percentages of CD4+ IL-17A+ T cells and CD4+ CD25+ FOXP3+ T cells in lung tissues from each group were determined using flow cytometry. (**O**–**Q**) RORγt and FOXP3 expression in lung tissue was analyzed using Western blots. Data are presented as means ± SDs and analyzed using unpaired *T* tests. *n* = 3–6 for each group. * *p* < 0.05, ** *p* < 0.01, and *** *p* < 0.001 indicate significant differences from each group. SPX, splenectomy.

The vagus nerve senses inflammation and regulates the immune response by controlling key immune organs. The spleen is the largest immune organ in the body and plays a central role in the inflammatory response [28]. Previous studies have shown that the spleen plays an important role in the anti-inflammatory effects of VNS [5]. To evaluate whether the spleen mediates VNS-induced regulation of Th17/Treg homeostasis and lung protection in ARDS model rats, splenectomy was performed 7 days prior to LPS tracheal instillation. H&E staining revealed significantly increased lung damage in the SPX + LPS + VNS group compared with the LPS + VNS group, with markedly higher pathological scores (Figure 4A,B). Consistently, the lung wet-to-dry (W/D) ratio was significantly elevated (Figure 4C). Furthermore, total protein levels of pro-inflammatory cytokines (TNF-α and IL-1β) in BALF were significantly increased in the SPX + LPS + VNS group (Figure 4D–F). The levels of Th17-related cytokines (IL-6, IL-17, and IL-23) were also elevated, while the levels of Treg-associated cytokines (IL-2 and IL-10) were significantly reduced (Figure 4G–K). Flow cytometry and Western blot analyses showed a significant increase in the proportion of Th17 cells and RORγt expression, along with a decrease in Treg cells and FOXP3 expression in lung tissue (Figure 4L–Q). These findings suggest that VNS potentially alleviates lung injury and regulates Th17/Treg homeostasis in ARDS model rats through mechanisms involving the spleen.

### 3.5. VNS Upregulates Splenic α7nAChR Expression and Modulates Splenic Th17/Treg Homeostasis in a Rat Model of ARDS

**Figure 5:**
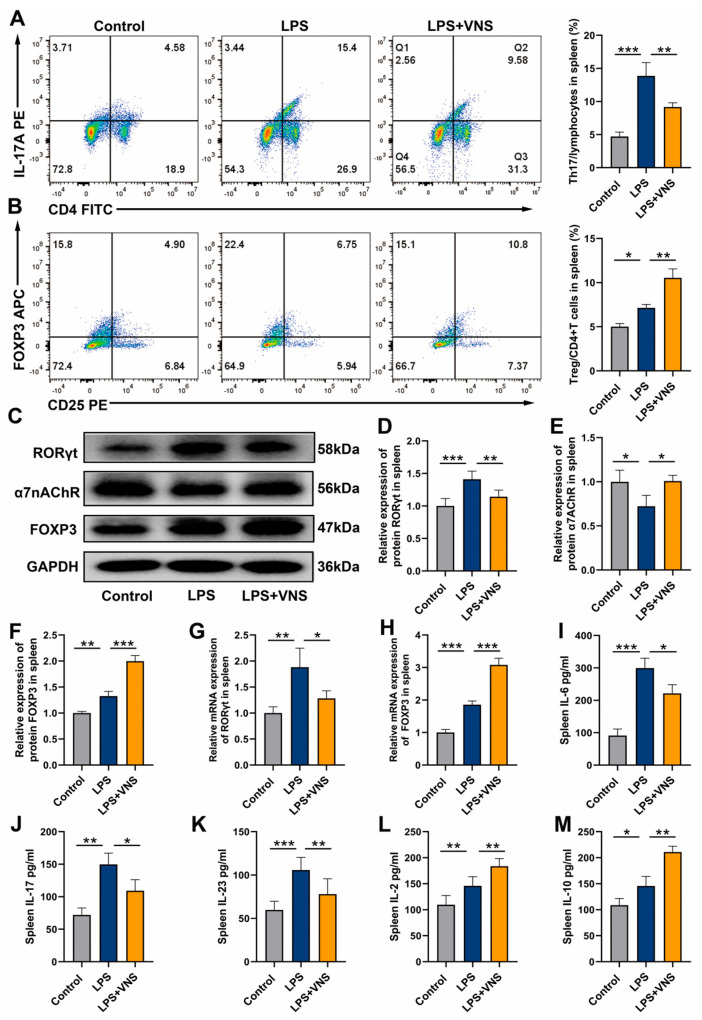
(**A**,**B**) The percentages of CD4+ IL-17A+ T cells and CD4+ CD25+ FOXP3+ T cells in each group of spleen tissue were determined using flow cytometry. (**C**–**F**) RORγt, α7nAChR, and FOXP3 expression in spleen tissues was analyzed using Western blots. (**G**,**H**) RORγt and FOXP3 mRNA levels in spleen tissue were measured using qRT-PCR. (**I**–**M**) The level of inflammatory cytokines IL-6, IL-17, IL-23, IL-2, and IL-10 in the spleen as assessed using ELISA. Data are presented as means ± SDs and analyzed using one-way ANOVA followed by the Bonferroni’s post-hoc test. *n* = 3–6 for each group. * *p* < 0.05, ** *p* < 0.01, and *** *p* < 0.001 indicate significant differences from each group.

Our findings indicated that the spleen plays a key role in VNS by alleviating the inflammatory response and regulating Th17/Treg homeostasis in ARDS model rats. Based on these observations, we postulate that some proteins or cells in the spleen are involved in the protection of VNS against ARDS. In the classical CAIP, the α7nAChR is a key protein in the spleen. The vagus nerve activates it by releasing acetylcholine (ACh), thereby reducing the inflammatory response [5]. Therefore, we examined Th17, Treg, and α7nAChR expression in the spleen. Analysis of splenocytes revealed that, compared to the control group, LPS treatment increased the percentage of Th17 cells and decreased the percentage of Treg cells (Figure 5A,B). Western blot analysis showed that LPS treatment significantly upregulated RORγt and FOXP3 expression levels in spleen tissues but caused significant downregulation of α7nAChR protein expression (Figure 5C–F). Notably, VNS intervention reversed the above changes, downregulating RORγt expression levels, while significantly upregulating FOXP3 and α7nAChR expression levels (Figure 5C–F). Consistent with this, qRT-PCR assay results showed that RORγt and FOXP3 mRNA expression levels were significantly increased in the LPS-treated group compared to the control group. In contrast, VNS treatment significantly inhibited the transcriptional activity of RORγt and further promoted the expression of FOXP3 mRNA (Figure 5G,H). ELISA analysis of spleen tissue homogenates revealed that VNS reduced Th17-related cytokines IL-6, IL-17, and IL-23 but increased Treg-related cytokines IL-2 and IL-10 (Figure 5I,M). These findings indicate that VNS alleviates the inflammatory response in ARDS, likely through α7nAChR activation in the spleen and the modulation of splenic Th17/Treg homeostasis.

### 3.6. VNS Regulates Th17/Treg Homeostasis and Attenuates LPS-Induced ARDS Through α7nAChR Activation in the Spleen

**Figure 6:**
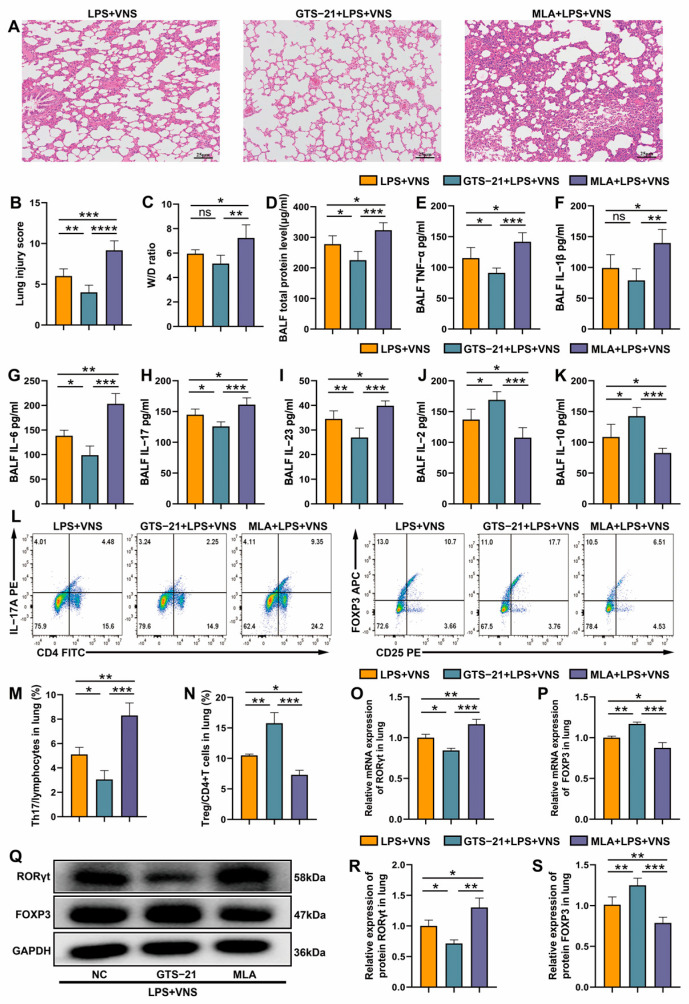
(**A**) Representative images of H&E staining (100× magnification) of lung tissue sections from each group. (**B**) Lung injury score analysis. (**C**) The wet-to-dry ratio of lung tissues. (**D**) Protein concentrations in BALF. (**E**–**K**) The levels of inflammatory cytokines TNF-α, IL-1β, IL-6, IL-17, IL-23, IL-2, and IL-10 in BALF as assessed using ELISA. (**L**–**N**) The percentages of CD4+ IL-17A+ T cells and CD4+ CD25+ FOXP3+ T cells in lung tissues from each group were determined using flow cytometry. (**O**,**P**) RORγt and FOXP3 mRNA expression in lung tissue was measured using qRT-PCR. (**Q**–**S**) RORγt and FOXP3 expression in lung tissues were analyzed using Western blots. Data are presented as means ± SDs and analyzed using one-way ANOVA followed by the Bonferroni’s post-hoc c test. *n* = 3–6 for each group. * *p* < 0.05, ** *p* < 0.01, and *** *p* < 0.001, **** *p* < 0.0001 indicate significant differences from each group. NC, negative control; GTS-21, α7nAChR agonist; MLA, α7nAChR antagonist.

Previous studies have shown that α7nAChR activation alleviates lung injury [29]. Furthermore, we found that VNS upregulates splenic α7nAChR protein expression. Therefore, we sought to investigate the impact of α7nAChR upregulation and downregulation on the ability of VNS to mitigate lung injury and regulate Th17/Treg homeostasis in ARDS model rats. Pathological assessment with H&E staining showed that the GTS-21 (α7nAChR agonist) preconditioned group exhibited significantly reduced lung histopathological injury and lower lung injury scores compared with the LPS + VNS group, whereas the MLA (α7nAChR antagonist) preconditioned group showed a trend toward increased injury and higher lung injury scores (Figure 6A,B). The lung tissue W/D ratio did not differ between the GTS-21 preconditioned group and the LPS + VNS group, whereas MLA preconditioning significantly increased the degree of lung edema (Figure 6C). To investigate the role of the α7nAChR pathway in the regulation of VNS, we systematically evaluated relevant inflammatory indicators. Bronchoalveolar lavage fluid (BALF) analysis showed that GTS-21 pretreatment significantly decreased total protein concentrations and TNF-α levels, whereas MLA pretreatment significantly increased total protein concentrations as well as TNF-α and IL-1β levels compared with the LPS + VNS group (Figure 6D–F). In terms of VNS regulation of Th17/Treg homeostasis, compared with the LPS + VNS group, GTS-21 intervention significantly downregulated the expression of Th17-associated cytokines IL-6, IL-17, and IL-23 but upregulated the levels of Treg-associated factors IL-2 and IL-10. Moreover, MLA intervention showed the completely opposite regulatory trend (Figure 6G–K). Flow cytometry analysis showed that GTS-21 pretreatment decreased the proportion of Th17 cells, increased the proportion of Treg cells, and decreased the Th17/Treg ratio in lung tissue compared with the LPS + VNS group. In contrast, MLA pretreatment significantly increased the proportion of Th17 cells, decreased the proportion of Treg cells, and increased the Th17/Treg ratio (Figure 6L–N). The qRT-PCR and Western blot results further confirmed that GTS-21 pretreatment significantly inhibited RORγt expression and increased FOXP3 expression compared with the LPS + VNS group, and the opposite result was noted with MLA pretreatment (Figure 6O–S). These results suggested that VNS regulates the lung Th17/Treg balance and attenuates lung injury in ARDS model rats through α7nAChR activation.

### 3.7. The Regulation of Splenic Th17/Treg Homeostasis by VNS Is α7nAChR Dependent

**Figure 7:**
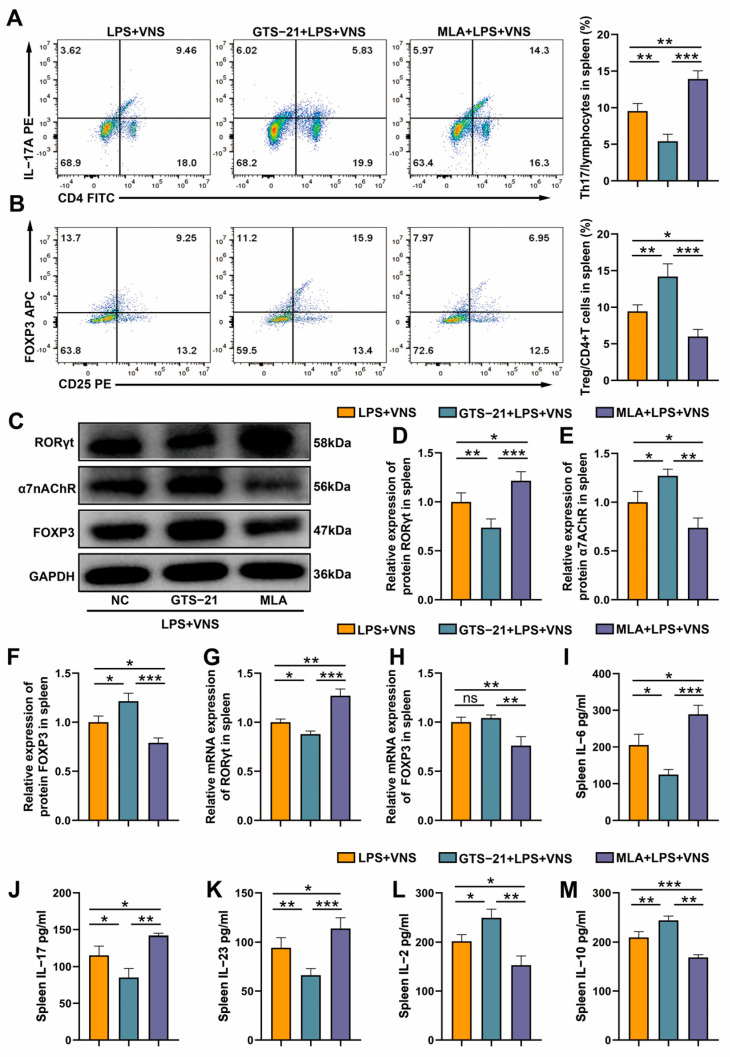
(**A**,**B**) The percentage of CD4+ IL-17A+ T cells and CD4+ CD25+ FOXP3+ T cells in spleen tissues from each group were determined using flow cytometry. (**C**–**F**) RORγt, α7nAChR, and FOXP3 expression in spleen tissues was analyzed using Western blots. (**G**,**H**) The mRNA of RORγt and FOXP3 in spleen tissue was measured by qRT-PCR. (**I**–**M**) The levels of inflammatory cytokinesIL-6, IL-17, IL-23, IL-2, and IL-10 in the spleen as assessed using ELISA. Data are presented as means ± SDs and analyzed using one-way ANOVA followed by the Bonferroni’s post-hoc test. *n* = 3–6 for each group. * *p* < 0.05, ** *p* < 0.01, and *** *p* < 0.001 indicate significant differences from each group. NC, negative control; GTS-21, α7nAChR agonist; MLA, α7nAChR antagonist.

Flow cytometry analysis showed that GTS-21 pretreatment decreased the proportion of Th17 cells, increased the proportion of Treg cells, and decreased the Th17/Treg ratio in splenic tissues compared with the LPS + VNS group. In contrast, the results of MLA pretreatment were completely opposite to those of GTS-21 pretreatment (Figure 7A,B). Western blot analysis showed that, compared with the LPS + VNS group, GTS-21 pretreatment significantly inhibited RORγt expression while enhancing FOXP3 and α7nAChR expression in spleen tissues, whereas MLA pretreatment showed completely opposite results (Figure 7C–F). The qRT-PCR analysis showed that, compared to the LPS + VNS group, GTS-21 pretreatment significantly decreased RORγt mRNA expression in spleen tissues, whereas MLA pretreatment increased RORγt mRNA expression and decreased FOXP3 mRNA expression (Figure 7G,H). ELISA of spleen tissue homogenates showed that GTS-21 pretreatment significantly downregulated the expression of Th17-related cytokines IL-6, IL-17, and IL-23 and upregulated the levels of Treg-related factors IL-2 and IL-10 compared to the LPS + VNS group, while MLA intervention showed a completely opposite regulatory trend (Figure 7I–M). These results suggest that VNS regulates Th17/Treg homeostasis in the spleen through α7nAChR activation.

### 3.8. VNS Modulates Inflammatory Factors in the Serum of LPS-Induced ARDS Model Rats

**Figure 8:**
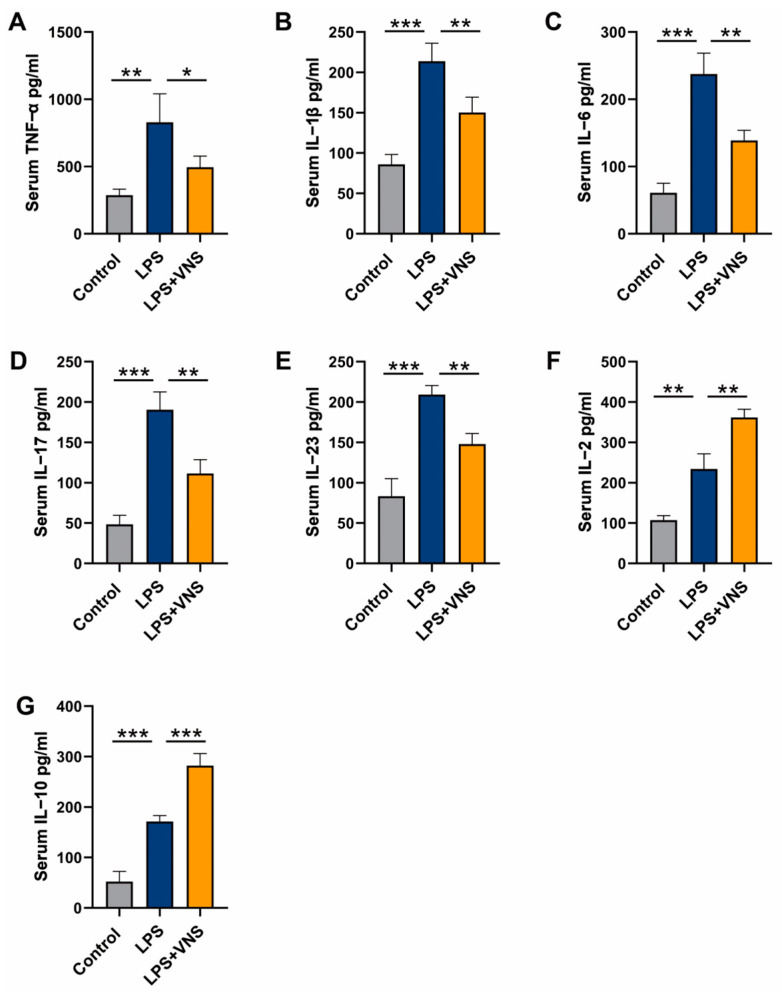
(**A**–**G**) The levels of inflammatory cytokines TNF-α, IL-1β, IL-6, IL-17, IL-23, IL-2, and IL-10 in serum as assessed using ELISA. Data are presented as means ± SDs and analyzed using one-way ANOVA followed by the Bonferroni’s post-hoc test. *n* = 3–6 for each group. * *p* < 0.05, ** *p* < 0.01, and *** *p* < 0.001 indicate significant differences from each group.

We measured the levels of inflammatory factors in the serum and found that, compared to the LPS treatment group, VNS reduced the levels of pro-inflammatory cytokines TNF-α and IL-1β as well as Th17-related cytokines IL-6, IL-17, and IL-23 (Figure 8A–E). In addition, VNS increased the levels of Treg-related cytokines IL-2 and IL-10 (Figure 8F,G). These findings suggest that the ability of VNS to alleviate lung injury and regulate Th17/Treg homeostasis in ARDS model rats is potentially related to the levels of inflammatory factors.

## 4. Discussion

This study explored whether VNS reduced pulmonary inflammation in ARDS by regulating Th17/Treg homeostasis. Our results demonstrated that VNS effectively regulates Th17/Treg homeostasis, thereby attenuating the inflammatory response in the lungs of LPS-induced ARDS model rats. This regulatory effect was mediated through activation of the CAIP, independently of the HPA axis. Both afferent and efferent vagal stimulation exerted comparable anti-inflammatory effects, likely due to their shared activation of vagal preganglionic neurons.

Studies have reported that VNS alleviates lung injury in ARDS by activating α7nAChRs in the CAIP, thereby influencing macrophage polarization [7]. Our previous research demonstrated that modulating Th17/Treg homeostasis alleviates lung injury in ARDS [30]. Building on these findings, we first investigated the regulatory effects of VNS on Th17/Treg homeostasis. In LPS-induced ARDS rat models, we found that VNS intervention improved lung pathology, reduced inflammatory cytokine levels, and decreased the Th17/Treg ratio. Consistently, our results indicated that VNS attenuates pulmonary inflammation in ARDS by regulating Th17/Treg homeostasis. VNS activates both afferent (sensory) and efferent (motor) fibers of the vagus nerve, with afferent fibers comprising approximately 80% and efferent fibers about 20% of total vagal fibers [27]. Previous studies have shown that the anti-inflammatory effects of afferent and efferent VNS vary across different inflammatory disease models. For example, in models of acute kidney injury and endotoxemia, both afferent and efferent VNS reduced inflammation and improved tissue injury [31,32,33]. However, in experimental arthritis, VNS regulates joint inflammation primarily through a central nervous system-mediated afferent vagus pathway, whereas efferent VNS had no significant effect [34]. In the ARDS model rats, we found that both afferent and efferent VNS were comparable to intact VNS in alleviating lung injury and regulating Th17/Treg homeostasis. These differences may reflect the distinct mechanisms by which VNS exerts its effects in different disease contexts.

The anti-inflammatory pathway of the HPA axis is initiated by the activation of afferent vagus nerves by systemic inflammation. This activation transmits signals via adrenergic fibers to the hypothalamus through the nucleus tractus solitarius. Subsequently, under the regulation of the pituitary gland, the pathway ultimately stimulates the adrenal cortex to release glucocorticoids, thereby exerting anti-inflammatory effects [8,35]. We found that the adrenal gland does not contribute to VNS-mediated attenuation of pulmonary inflammation and regulation of the Th17/Treg balance in ARDS model rats. Consistent with previous studies demonstrating that the anti-inflammatory effects of VNS are dependent on the spleen rather than the adrenal gland, our findings support this conclusion [5]. Previous studies have shown that both afferent and efferent VNS inhibit the release of inflammatory factors by activating splenic nerves and spleen cells [32,33]. The spleen also plays a key role in the vagal regulation of peripheral inflammation [4,28]. In models of kidney injury and colitis, studies have demonstrated that the anti-inflammatory effects of VNS are dependent on spleen involvement [31,36]. However, in models of acute pancreatitis and gastrointestinal inflammation, the spleen does not appear to be involved in VNS-mediated anti-inflammatory effects [37,38]. In our study, we observed that in ARDS model rats, the spleen plays a role in mediating the effects of VNS in alleviating pulmonary inflammation and regulating Th17/Treg homeostasis. This finding may reflect the diversity of the anti-inflammatory pathways activated by VNS. The vagus nerve exerts its anti-inflammatory effects through both the classical and non-classical CAIP. In the classical pathway, VNS indirectly activates α7nAChR^+^ macrophages in the spleen via the splenic nerves, thereby alleviating inflammation [6,31]. In contrast, the non-classical pathway involves direct activation of α7nAChR-positive macrophages in target organs by VNS to reduce inflammation [37].

The α7nAChR is a key protein in the CAIP and plays a crucial role in inhibiting inflammasome activation [4,39]. The vagus nerve specifically controls splenic noradrenergic fibers via the α7nAChR, and the splenic nerve can suppress cytokine production in splenic macrophages [5,6]. Moreover, VNS did not exert peripheral anti-inflammatory functions in α7nAChR knockout mice [40]. Studies in a rat model of irritable bowel syndrome demonstrated that both α7nAChR upregulation and downregulation modulate the anti-inflammatory effects of VNS [41]. Our research revealed that α7nAChR protein expression was reduced in the spleens of LPS-induced ARDS model rats, but was upregulated following VNS intervention. We hypothesized that the capacity of VNS to regulate the Th17/Treg balance and alleviate lung inflammation in ARDS rats may be related to splenic α7nAChR expression. Following the administration of the α7nAChR agonist GTS-21, VNS-mediated regulation of Th17/Treg homeostasis and reduction in inflammatory cytokine levels were further enhanced. In contrast, administration of the α7nAChR antagonist MLA suppressed these effects of VNS. These findings suggest that VNS regulates Th17/Treg homeostasis and mitigates lung inflammation in ARDS rats by upregulating splenic α7nAChR expression.

We demonstrated that VNS alleviates excessive inflammatory responses in the lungs of ARDS model rats by modulating Th17/Treg homeostasis both in the lungs and the spleen. However, the relationship between spleen and lung Th17/Treg cells as well as the mechanisms by which spleen Th17 and Treg cells influence pulmonary inflammation remain unclear. Previous studies have reported that VNS improves lung injury by inhibiting the release of α7nAChR^+^ CD11b^+^ cells from the spleen, thereby limiting their migration to the lungs [42]. This finding suggests that splenic immune cells may mitigate pulmonary inflammation by migrating to the lungs. Treg cells are predominantly found in peripheral blood and lymphoid tissues but they could selectively migrate to sites that require regulation, with accumulation observed at sites of infection and inflammation [43,44].

Furthermore, previous studies have demonstrated that regression of acute lung injury is associated with BTL1-dependent aggregation of Treg cells in the alveoli [12]. Therefore, we hypothesize that splenic Treg cells may migrate to the lungs using a specific pathway, thereby alleviating the hyperinflammatory response. In the present study, we experimentally observed a significant concordance between the trends in Th17-related and Treg-related cytokines in the spleen and serum following VNS intervention in a lipopolysaccharide (LPS)-induced ARDS rat model, suggesting a potential mechanism whereby splenic-derived Treg cells potentially migrate to lung tissues via the circulation. However, because circulating Treg cells were not directly measured, this inference requires further validation. Notably, Treg cells from lymphoid organs other than the spleen may also participate in the pulmonary immunoregulatory network. Previous studies have demonstrated that the bone marrow acts as an important reservoir for human Treg cells, mediating their dynamic balance with peripheral tissues via the CXCL12/CXCR4 signaling axis [45]. During immune responses, Treg cells are programmed to migrate, establishing regulatory loops between inflammatory sites and draining lymph nodes. In the context of the present study, the precise origin of Treg cells recruited to the lungs remains unclear and may involve complex mechanisms of multiorgan synergy [46,47]. Therefore, future research should focus on promoting or inhibiting Treg cell recruitment to the lungs. Our findings indirectly suggest that VNS may facilitate lung Treg cell recruitment, thereby attenuating inflammatory injury in ARDS rats.

Previous studies have shown that the anti-inflammatory effects of VNS are primarily mediated by α7nAChR-positive macrophages [48]. For instance, VNS attenuates lung injury in ARDS by promoting macrophage polarization from the pro-inflammatory M1 phenotype to the anti-inflammatory M2 phenotype [7]. Although this process appears to be independent of Th17 and Treg cells, there is an interaction between Treg cells and macrophages. Treg cells are capable of inhibiting macrophage polarization toward the M1 phenotype while promoting polarization toward the M2 phenotype [49,50]. Similarly, the neutralization of high-mobility group box 1 (HMGB1) or the knockdown of phosphatase and tensin homolog (PTEN) in macrophages increases Treg cells and decreases Th17 cells, thereby attenuating the inflammatory response [51]. Additionally, pulmonary macrophages in the lungs are thought to modulate Treg cell activity, thereby contributing to the resolution of lung inflammation [52]. Inflammatory cell apoptosis is the basis for the regression of inflammation, and Treg cells enhance macrophage phagocytosis [50]. Moreover, macrophages secrete cytokines that regulate the progression of inflammation, while cytokine signaling is crucial for the differentiation and function of both Th17 and Treg cells. Therefore, we hypothesize that VNS-mediated regulation of Th17/Treg homeostasis via α7nAChR to attenuate inflammation potentially involves macrophages.

A major limitation to the clinical application of VNS is its invasive nature. However, recent advancements have enabled immunomodulation with transcutaneous auricular vagus nerve stimulation (taVNS). Recent studies using rat models of gastrointestinal disease have found that taVNS upregulates intestinal ACh and α7nAChR expression, while simultaneously reducing the levels of inflammatory cytokines such as TNF-α, IL-1β, and IL-6 [53]. In the future, taVNS may serve as a potential therapeutic strategy for ARDS. In conclusion, our study demonstrates that VNS alleviates lung inflammation in ARDS model rats by upregulating splenic α7nAChR expression to modulate the Th17/Treg balance. These findings reveal a novel mechanism by which VNS alleviates ARDS and provide a theoretical basis for its therapeutic application.

## Data Availability

All data generated or analyzed during the present study are included in this article. Further inquiries can be directed to the corresponding author.

## Abbreviations

The following abbreviations are used in this manuscript:

ARDS	acute respiratory distress syndrome
VNS	vagus nerve stimulation
BALF	bronchoalveolar lavage fluid
LPS	lipopolysaccharide
HPA	hypothalamic–pituitary–adrenal axis
α7nAChR	A7 nicotinic acetylcholine receptor
Treg	regulatory T cell
Th17	helper T 17 cell
IL-1β	interleukin-1β
IL-2	interleukin-2
IL-6	interleukin-6
IL-10	interleukin-10
IL-17	interleukin-17
IL-23	interleukin-23
TNF-α	transforming growth factor-α
RORγt	retinoic acid receptor-related orphan receptor gamma t
FOXP3	forkhead box protein 3
W/D	wet-to-dry ratio
SPX	splenectomy
ADX	adrenalectomy
